# Phytolith Analysis for Differentiating between Foxtail Millet (*Setaria italica*) and Green Foxtail (*Setaria viridis*)

**DOI:** 10.1371/journal.pone.0019726

**Published:** 2011-05-06

**Authors:** Jianping Zhang, Houyuan Lu, Naiqin Wu, Xiaoyan Yang, Xianmin Diao

**Affiliations:** 1 Key Laboratory of Cenozoic Geology and Environment, Institute of Geology and Geophysics, Chinese Academy of Sciences, Beijing, China; 2 Institute of Geographic Sciences and Natural Resources Research, Chinese Academy of Sciences, Beijing, China; 3 The National Key Facility for Crop Gene Resources and Genetic Improvement, Institute of Crop Science, Chinese Academy of Agricultural Sciences, Beijing, China; University of Oxford, United Kingdom

## Abstract

Foxtail millet (*Setaria italica*) is one of the oldest domesticated cereal crops in Eurasia, but identifying foxtail millets, especially in charred grains, and differentiating it from its wild ancestor, green foxtail (*Setaria viridis*), in the archaeobotanical remains, is still problematic. Phytolithic analysis provides a meaningful method for identifying this important crop. In this paper, the silicon structure patterns in the glumes, lemmas, and paleas from inflorescence bracts in 16 modern plants of foxtail millet and green foxtail from China and Europe are examined using light microscopy with phase-contrast and a microscopic interferometer. Our research shows that the silicon structure of ΩIII from upper lemmas and paleas in foxtail millet and green foxtail can be correspondingly divided into two groups. The size of ΩIII type phytolith of foxtail millet is bigger than that from green foxtail. Discriminant function analysis reveals that 78.4% of data on foxtail millet and 76.9% of data on green foxtail are correctly classified. This means certain morphotypes of phytoliths are relatively reliable tools for distinguishing foxtail millet from green foxtail. Our results also revealed that the husk phytolith morphologies of foxtail millets from China and Eastern Europe are markedly different from those from Western Europe. Our research gives a meaningful method of separating foxtail millet and green foxtail. The implications of these findings for understanding the history of foxtail millet domestication and cultivation in ancient civilizations are significant.

## Introduction

Understanding the process of plant domestication is fundamental to our comprehension of the rise of agriculture [1]. Plant domestication involves a series of profound changes (morphologic and genetic) resulting from selection that make wild species more amenable to cultivation and consumption by humans [2]–[4]. Therefore, how to distinguish cultivated grain crops from their closely related wild grasses from archaeobotanical remains is essential for understanding the origin and spread of agriculture.

In North China, millet was the principal crop in the Neolithic period [5]–[7]. Millets served as the staple grains that allowed Chinese agricultural civilization to flourish in ancient times. To date, the issues about where, when and how the crop transitioned from simple gathering to domestication are still unclear [6], [8], [9], thus the necessity to distinguish foxtail millet (*Setaria italica*) from its ancestor, green foxtail (*Setaria viridis*), in archaeological remains.

For example, although foxtail millet-like residues have been found at sites dated to the early Holocene across a broad belt of Eurasia [9]–[15], the geographic origin of foxtail millet is a controversial issue. Many hypotheses have been suggested, such as the North China center [16], [17], south-central Asia center [18], central Europe center [19], [20], and multiple centers [21]–[26], although there has not been sufficient evidence to support any one of these hypotheses. The problem is further complicated due to an inability to distinguish foxtail millet from green foxtail in archaeological remains during the early stages of domestication. One important line of inquiry to differentiate the two is to study the grain or inflorescence bract morphology of foxtail millet and green foxtail.

To accomplish this task is difficult. First of all, because the grain size of both foxtail millet and green foxtail is very small and their grain shapes are similar [27], [28], researchers are likely to confuse the two in the archaeological remains. A previous study considered that the overlapping range of grain length to breadth ratios between foxtail millet and green foxtail is an obstacle for identification of these two species [29]. Second, in some sites, macro-remains are oxidized by heat into granules or ash, thus losing critical diagnostic features [13], [28]. The morphology of starch grains also has been considered as a method to distinguish between them [30]–[32], but it has not yet been confirmed.

Phytoliths are silica casts of plant cells created within and between cells of living plants tissues that can remain in sediments long after the living tissue has decayed [33]. Since phytoliths are replicas of plant cell bodies, they can be used to identify a certain genus or species according to shape, size, and other anatomical features [34]–[37]. The development and application of phytolith techniques make them useful tools for revealing historic vegetation patterns and human uses across the fields of archaeology, palaeoethnobotany, palaeoecology, and historical ecology in sites where preservation of macrobotanical remains is poor [13], [35], [38]–[45].

Distinguishing wild and domesticated species in archaeological residues in terms of phytolith morphology is a topic of great scientific interest [46], [47]. A number of phytolith studies have been conducted to focus on this problem, e.g., Piperno used morphological criteria to distinguish between Maize and wild grass phytoliths [43], Pearsall *et al.* and Zhao *et al.* used morphometric analysis to distinguish between domesticated and wild *Oryza* phytoliths [39], [44], Rosen *et al.*, Ball *et al.*, and Tubb *et al.* investigated phytolith morphology and the taxonomy of wheat, barley, and related wild grasses and offered preliminary methods to distinguish among them in archaeological samples [41], [48]–[52]. Lu *et al.* found five key phytolith diagnostic characteristics to distinguish common millet (*P. miliaceum*) from foxtail millet (*S. italica*), but did not include details about how to distinguish foxtail millet from green foxtail in archaeological remains [47]. To date, few studies have been conducted to differentiate the phytoliths of foxtail millet and green foxtail [53], and none thoroughly investigated the effect of domestication of foxtail millet on phytolith morphometry [47], [53].

In this paper, we attempt to determine if phytolith analysis of inflorescence bracts can be used as an effective tool for discriminating foxtail millet from green foxtail. Furthermore, based on modern millet samples from Europe and China, we discuss the origin of foxtail millet domestication. Our findings yield insights into the process of foxtail millet domestication across the semi-arid region of East Asia.

## Materials and Methods

**Figure 1:**
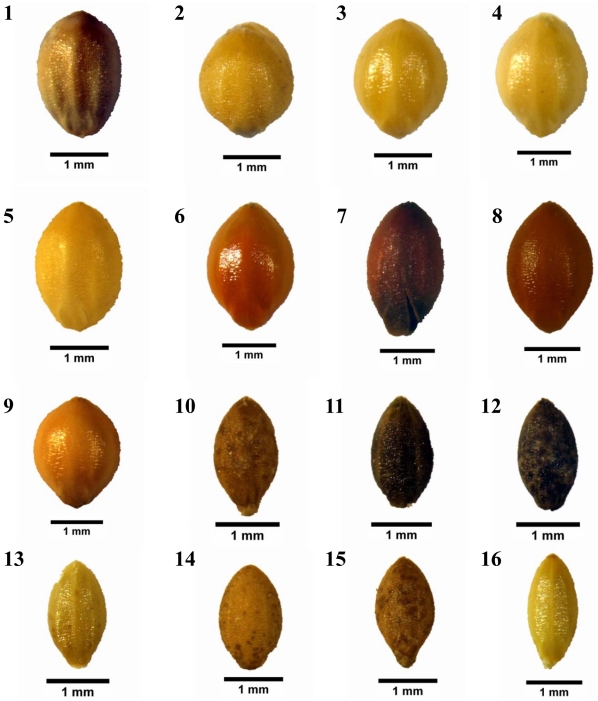
Photographs of *Setaria* grains. 1 = Z668, 2 = Z335, 3 = Z399, 4 = Z557, 5 = Z280, 6 = Z169, 7 = Z737, 8 = Z734, 9 = W28, 10 = Qing24, 11 = Qing68, 12 = Qing44, 13 = Qing46, 14 = Qing28, 15 = Qing7-1, 16 = Qing59.

We examined phytoliths from 9 samples of modern foxtail millet (*S. italica*) and 7 samples of green foxtails (*S. viridis*) obtained from annotated folders at the Institute of Millet Crops (IMC), Hebei Academy of Agriculture and Forestry Sciences, Shijiazhuang, China. The folders contained samples from field collections by many investigators. For detailed data on the plants, see Table 1 and Figure 1.

**Table 1:** Information on the plants studied.

No.	Species	IMC no.	Province/country of origin
S1	*Setaria italica* (L.) Beauv.	Z280	Gansu
S2	*Setaria italica* (L.) Beauv.	Z335	Jilin
S3	*Setaria italica* (L.) Beauv.	Z399	Henan
S4	*Setaria italica* (L.) Beauv.	Z502	Inner Mongolia
S5	*Setaria italica* (L.) Beauv.	Z557	Hebei
S6	*Setaria italica* (L.) Beauv.	W28	France
S7	*Setaria italica* (L.) Beauv.	Z169	France
S8	*Setaria italica* (L.) Beauv.	Z734	Hungary
S9	*Setaria italica* (L.) Beauv.	Z737	Romania
SV1	*Setaria viridis* (L.) Beauv.	Qing 7-1	Yunnan
SV2	*Setaria viridis* (L.) Beauv.	Qing 24	Hebei
SV3	*Setaria viridis* (L.) Beauv.	Qing 28	Hebei
SV4	*Setaria viridis* (L.) Beauv.	Qing 44	Liaoning
SV5	*Setaria viridis* (L.) Beauv.	Qing 46	Shanxi
SV6	*Setaria viridis* (L.) Beauv.	Qing 59	Ningxia
SV7	*Setaria viridis* (L.) Beauv.	Qing 68	Henan

**Figure 2:**
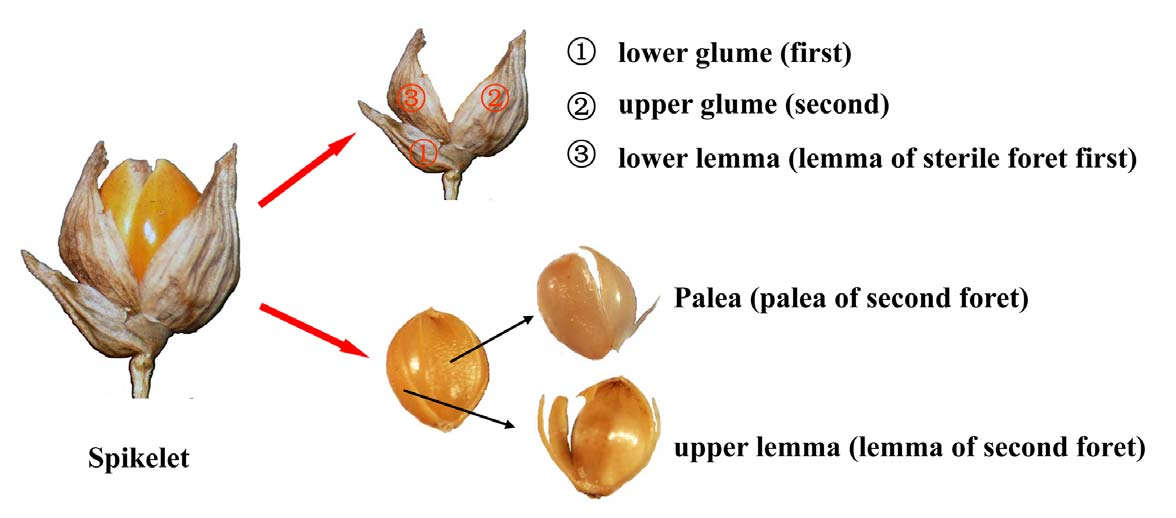
Illustrations of spikelet and grain of millets with botanical terms. After reference [47].

In this study, three parts were dissected from the spikelet of modern plants, including 1) lower glume, upper glume, and lower lemma (lemma of sterile floret), 2) upper lemma, and 3) palea for phytolith analysis (Figure 2) [29]. Palea can be divided into “palea of first floret” and “palea of second floret.” “Palea of first floret” atrophies into a very small membranous organ and sometimes becomes lost in the spikelet. Consequently, we used “palea” for the “palea of second floret” [47]. The three parts of the spikelet were prepared wet oxidation, as described by Lu *et al* [47].

## Results

### Phytolith morphology of glumes and lower lemmas

**Figure 3:**
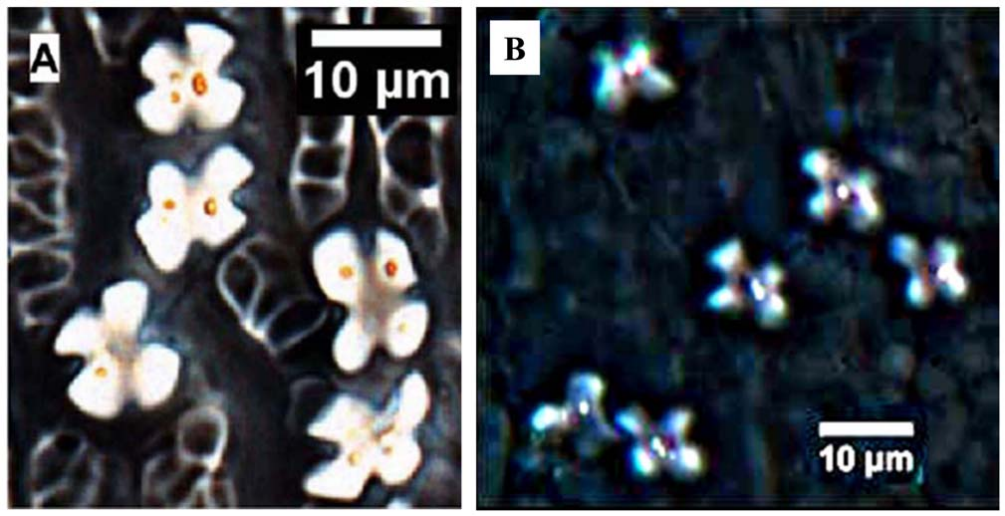
Comparison of phytolith morphology in the lower lemmas and glumes from foxtail millet and green foxtail. (A) Cross-shaped phytolith from foxtail millet, after reference [47], (B) Cross-shaped phytolith from green foxtail.

Silica can occur in many parts of plants, including cell walls, cell lumens, intracellular spaces, roots, and leaves [54]. For example, in foxtail millet, its silica deposition always occurs in short cells (silica bodies) of the caryopsis and in inflorescence bracts, such as glumes and lemmas [53]. Based on our observations and statistics, we found silica bodies from glumes and lower lemmas of foxtail millet and green foxtail were generally similar (Figure 3).

Cross-shaped (ratio of length to width ≈1∶1) phytoliths were found in glumes and lower lemmas of both foxtail millet and green foxtail. The size variation (length) tended to increase toward the central part of the lower lemmas and glumes (foxtail millet: range 4.46–9.98 µm, average 7.55±1.17 µm, n = 208; green foxtail: range 5.68–10.54 µm, average 7.67±0.86 µm, n = 201) [47].

A few silicified long cells, micro-hairs, macro-hairs, and stomata in the lower lemma and glumes of both taxa had no diagnostic character shape, so were not easily identified in terms of phytoliths. This suggests that the cross-shaped phytoliths formed in the lower lemmas and glumes cannot be used as criteria to distinguish foxtail millet from green foxtail.

### The undulating patterns of epidermal long cells in upper lemmas and paleas

**Figure 4:**
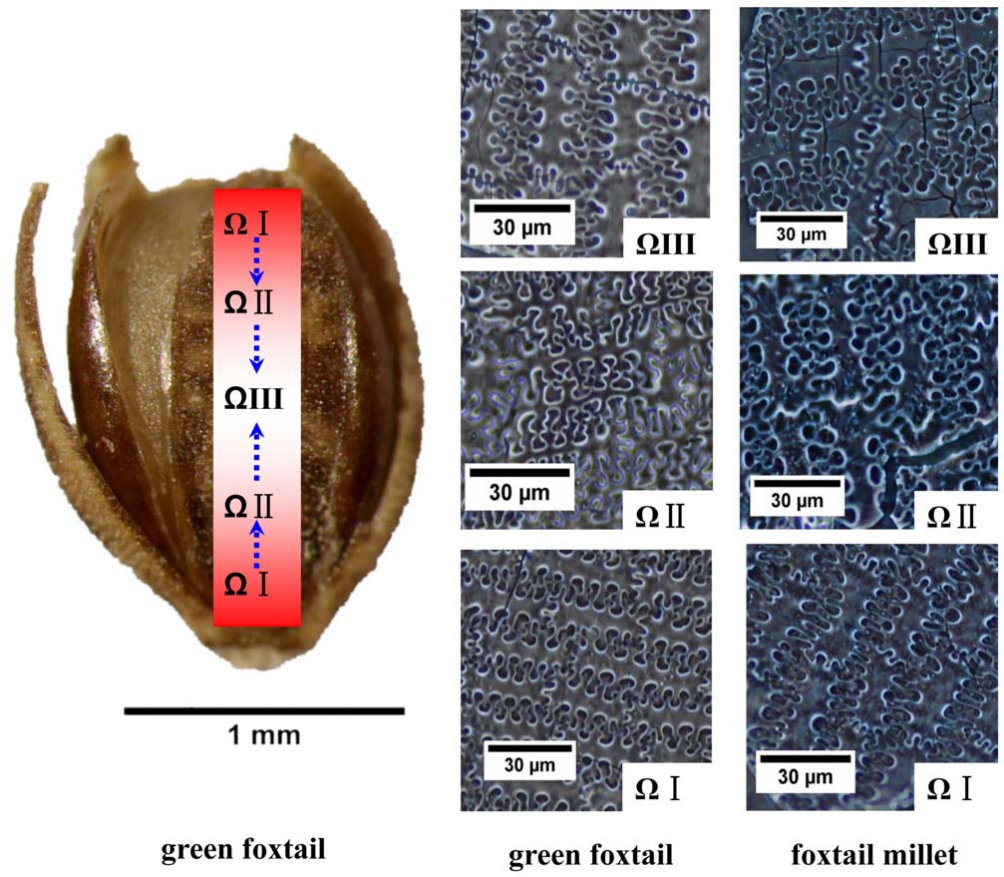
Comparison of undulated patterns of silicified epidermal long cells in different parts of the upper lemmas and paleas from foxtail millet and green foxtail.

The undulating patterns of epidermal long cells in the upper lemmas and paleas in green foxtail were complex and similar to those from foxtail millet, such as the Ω-type (which can be subdivided into ΩI, ΩII, and ΩIII) (Figure 4). Having similar undulations as foxtail millet, in green foxtail the undulations tend to increase in highly sinuous variation toward the central part of the lemmas and paleas. The different Ω-undulated patterns occur at different points by a gradual change from base to top (ΩI), to side (ΩII), and to center (ΩIII) of the lemmas and paleas in green foxtail (Figure 4).

**Figure 5:**
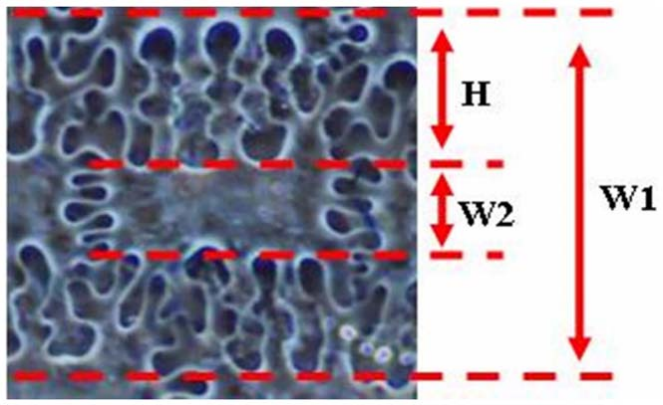
Parameters of undulated patterns of silicified epidermal long cells from foxtail millet and green foxtail.

According to our observations and statistics for structures of epidermal long cells, three important parameters can be used to define morphological variations between foxtail millet and green foxtail of structures of epidermal long cells in the upper lemmas and paleas: W1 = width of undulated patterns of epidermal long cells, W2 = width of epidermal long cells, H = undulation amplitude of dendriform epidermal long cell walls (Figure 5). The W1/H ratio was 2.53±0.17 (n = 1866) and 2.49±0.20 (n = 1457) in foxtail millet and green foxtail, respectively.

While it was not possible to distinguish them by relative W1/H ratio value of phytolith parameters, it was noteworthy that the absolute values of these parameters have taxonomic significance. We measured the values of W1, W2, and H1 according to the types of undulating patterns of epidermal long cell (ΩI, ΩII, and ΩIII), respectively. A Mann-Whitney U test [55] was performed for W1, W2 and H1 from foxtail millet and green foxtail (Table 2).

**Table 2:** The result of non-parametric test for W1, W2, and H of undulated patterns of epidermal long cells from foxtail millet and green foxtail.

Parameters	Species	No.	Mean rank	Mann-Whitney U	Z value	Asymp. Sig. (2-tailed)
W1-1	*S. italica*	537	517.63	73232.50	−7.56	0.000
	*S. viridis*	385	383.21			
W1-2	*S. italica*	457	485.25	95398.00	−2.69	0.007
	*S. viridis*	465	438.16			
W1-3	*S. italica*	872	926.52	102005.00	−20.13	0.000
	*S. viridis*	607	472.05			
W2-1	*S. italica*	537	495.97	84864.00	−4.64	0.000
	*S. viridis*	385	413.43			
W2-2	*S. italica*	457	513.81	82349.00	−5.91	0.000
	*S. viridis*	465	410.09			
W2-3	*S. italica*	872	960.06	72756.00	−23.75	0.000
	*S. viridis*	607	423.86			
H-1	*S. italica*	537	520.42	71733.50	−7.93	0.000
	*S. viridis*	385	379.32			
H-2	*S. italica*	457	476.38	99454.00	−1.68	0.093
	*S. viridis*	465	446.88			
H-3	*S. italica*	872	895.45	129103.50	−16.78	0.000
	*S. viridis*	607	516.69			

W(1,2)-Y(Y = 1,2,3): W1 = width of undulating patterns of epidermal long cells; W2 = width of epidermal long cells; H-X(X = 1,2,3 ) = undulation amplitude of dendriform epidermal long cell walls. X, Y = 1, 2, 3 = phytoliths in ΩI, ΩII, and ΩIII types.

The Mann-Whitney U test, a nonparametric test for comparison of two independent samples, is one of the most widely used nonparametric tests [56]. Unlike the parametric *t*-test, this non-parametric test makes no assumptions about the distribution of the data [57], but it can tell us whether or not the central tendencies (mean or median) of two groups were different from each other and can test whether or not two samples come from the same distribution based on the ranks of data of the two groups [56], [58]. We found that, except for parameter H-2, in all the samples, the Sig.<0.05, with a confidence level of 95%. This suggests that the data from type ΩIII phytoliths come from different distributions of foxtail millet and green foxtail.

**Figure 6:**
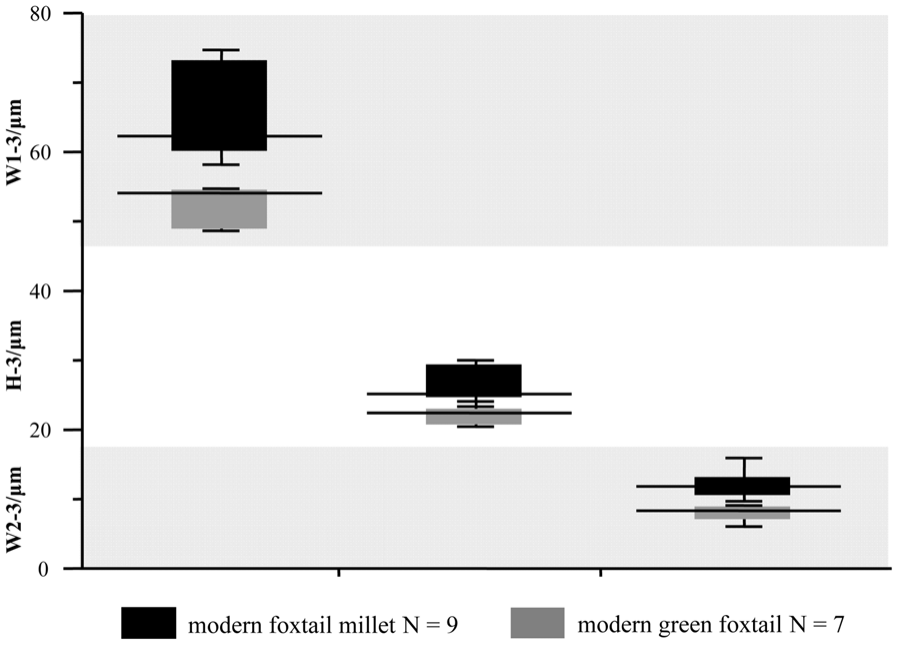
Comparison of W1-3, W2-3, and H-3 of undulating patterns of epidermal long cell from all *S. italica* and *S. viridis* based on Box Plot. 9 species for *S. italica* (872 data) and 7 species for *S. viridis* (607 data).

Figure 6 shows the average value of W1-3, W2-3, and H-3 in type ΩIII. The value of W1-3 was 64.2±5.9 µm in foxtail millet and 52.2±2.8 µm in green foxtail; W2-3 was 11.9±1.9 µm and 8.0±1.1 µm, and H-3 was about 26.1±2.2 µm and 22.1±1.4 µm, respectively. The ΩIII undulating patterns of epidermal long cells in the upper lemmas and paleas in foxtail millet were more extended than those in green foxtail. Based on these data, phytoliths from foxtail millet and green foxtail, while similar, can be somewhat distinguished from each other, and that only the ΩIII patterns of epidermal long cells in the upper lemmas and paleas can be used to statistically distinguish foxtail millet from green foxtail.

### Discriminant function analysis of upper lemmas and paleas

Discriminant function analysis is a statistical procedure for identifying boundaries between groups of objects by analyzing relationships of quantitative variables between these objects [59]. Discriminant function analysis is also applied to classify objects into respective groups by analyzing the relationships between variables of the objects and the boundaries defined in terms of these variables [39].

Discriminant analysis deals with three aspects of data: groups, objects, and variables. In this study, the species of foxtail millet and green foxtail can be regarded as two groups, the phytoliths of upper lemmas and paleas were the objects, and measurable characteristics of W1-3, W2-3, and H-3 were the variables (as they have the most significant difference between groups; see table 2). A total of 1479 data points (872 from foxtail millet and 607 from green foxtail) were used as the variables in this study. SPSS software was used for the discriminant analysis.

The results of the discriminant analysis are presented in Table 3 and as a percentage of correct classifications in Table 4. The within-group correlations between discriminating variables and standardized canonical discriminant functions variables are higher for W1-3 (0.958) and W2-3 (0.813), which means W1-3 and W2-3 contributed more to the discriminant function (Table 3); H-3 was not included in the function as it did not pass the tolerance test.

**Table 3:** Structure matrix and canonical discriminant function coefficients.

	W1-3	W2-3	H-3*	Constant
Structure matrix	0.958	0.813	0.638	/
Unstandardized coefficients	0.037	0.270	/	−4.925

*This variable not used in the analysis.

**Table 4:** Classification results of discriminant analysis for ΩIII type phytolith.

	Groups	Predicted group		Total
		*S. italica*	*S. viridis*	
Original count	*S. italica*	684	188	872
	*S. viridis*	140	467	607
%	*S. italica*	78.4	21.6	100.0
	*S. viridis*	23.1	76.9	100.0

78.4% of foxtail millet and 76.9% of green foxtail are classified accurately.

A total of 78.4% of upper lemmas and paleas were correctly classified into the foxtail millet group, and 76.9% into the green foxtail group (Table 4) for an average of 77.8% of the subjects correctly classified. These encouraging results indicated that the ΩIII characters of upper lemmas and paleas had a strong tendency to polarize between the foxtail millet and green foxtail.

**Figure 7:**
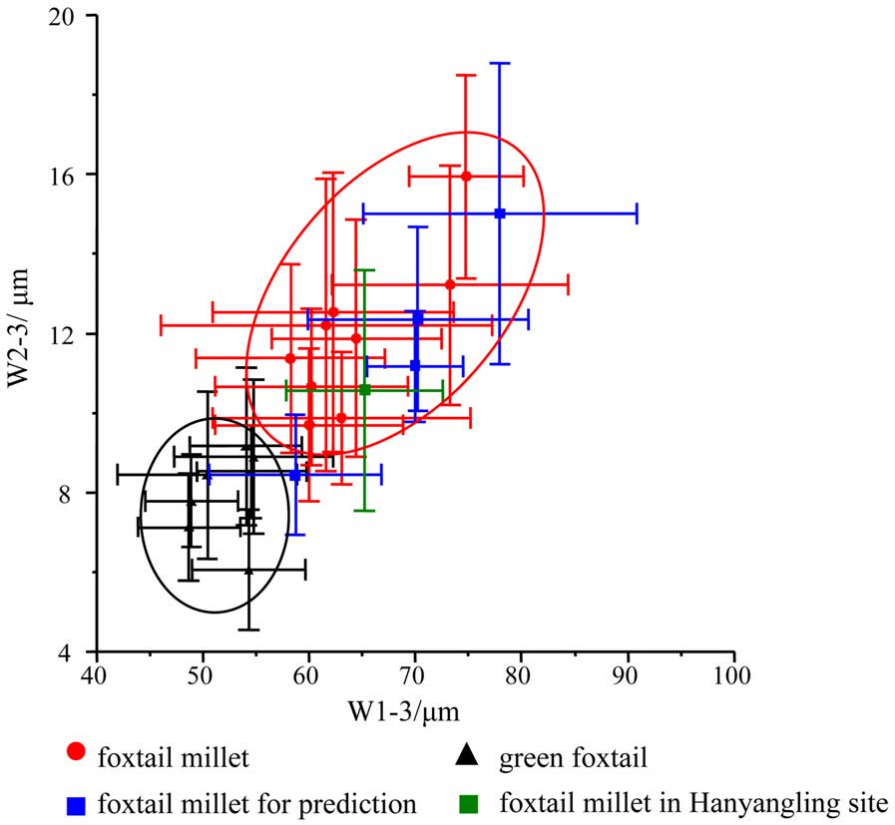
Bivariate biplot of W1-3 and W2-3 values of measurements from epidermal long cells of foxtail millet and green foxtail. W1-3 = width of undulated patterns of epidermal long cells of ΩIII; W2-3 = width of epidermal long cells of ΩIII. Error bar = SD.

Four varieties of modern foxtail millets, Shangdong dangdi gu, Gansu dangdi gu, Donghui gu, and Zhuyeqing gu, and one fossil sample of foxtail millet from the Han Yangling Mausoleum, built for an Emperor of the Han Dynasty, Liu Qi (188-141 BCE) and located to the north of Xi'an City, Shaanxi Province, China [60]. The five samples were selected at random to test the reliability of discriminant analysis. The mean values of 872 measurements from ΩIII epidermal long cells of foxtail millet (9 species) and 607 measurements from green foxtail (7 species) are shown in the bivariate biplot, plotted along axis W1-3 (width of undulating patterns of epidermal long cells in ΩIII) and axis W2-3 (width of epidermal long cells in ΩIII). They have been classified into two groups corresponding to the two species (foxtail millet and green foxtail) (Figure 7). The four varieties of modern foxtail millets and one fossil foxtail millet sample of husk phytoliths from Han Yangling Mausoleum are plotted for prediction. For each predicted sample, we calculate the mean value of 20 measurements for each ΩIII parameter (W1-3, W2-3, and H-3). As expected, Shangdong dangdi gu (from Shangdong Province, Eastern China) was situated in the transition zone between foxtail millet and green foxtail; the other three samples were all correctly classified.

### Phytolith papillae of upper lemmas and paleas

Phytolith papillae were found in both foxtail millet and green foxtail. While all green foxtail samples had regularly arranged papillae in upper lemmas and paleas. For Qing 59 from Ningxia (Northwest China) and Qing 46 from Shanxi (North Central China), however, only paleas have regularly arranged papillae; the silicified upper lemmas were smooth. Six foxtail millet samples had arranged papillae, but for Z734 from Hungary and Z557 from Hebei (North Central China), the papillae were weak and only present at the top and base of upper lemmas and paleas. For Z169 and W28 from France and Z399 from Henan (Central China), no regularly arranged papillae were found on upper lemmas or paleas.

**Figure 8:**
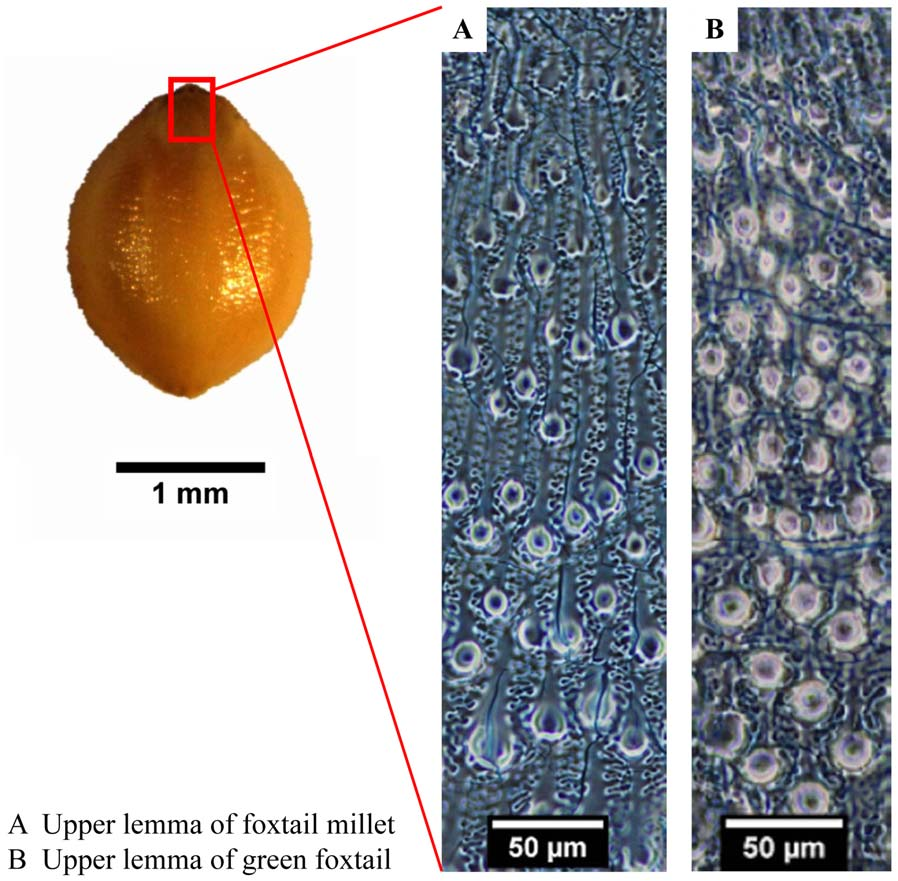
Papillae distribution on surfaces of the upper lemmas from foxtail millet and green foxtail.

The papillae from foxtail millet and green foxtail tend to have less size variation toward the base and top of the upper lemmas and paleas (Figure 8). Papillae diameters in foxtail millet range between 5 and 25 µm [47], whereas in green foxtail they range between 10 and 20 µm [29].

### The surface sculpture of epidermal long cells in upper lemmas

Different cell layers, such as extracellular sheet (keratose layer), outer epidermis, hypoderm fibers, vascular bundles, and spongy mesophyll, can be silicified in diverse patterns. We observed their morphology in the transections of lemmas and paleas [61].

The ridged lines of the surface sculpture consisted of a very heavy silicon layer that formed from an adnate silicon extracellular sheet and outer epidermis. Although this sculpture can be used to distinguish foxtail millet from common millet [47], based on our observations of surface characteristic with different adnate silicon layers in different Ω-types, the surface sculpture of the upper lemmas from foxtail millet was very similar to that from green foxtail. This means that the surface sculpture was not a good criterion to distinguish between these two species.

## Discussion

### Morphological difference in epidermal long cells between *S. italica* and *S. viridis*

Capturing domesticated trait alteration in morphology of plants (e.g., grain size, starch and phytolith shape) is particularly critical to understanding the transition from gathering to domestication. It is an effective way to document the emergence of domestication.

Early investigators have reported for foxtail millet, grain length-to-breadth ratios was 2.35-1.27, whereas in green foxtail, it was 3.27-1.76 [29]. A wider overlapping range of length-to-breadth ratios between these two species made it difficult to differentiate them.

Despite this wider overlapping range, on average foxtail millets have plumper grains than those of green foxtail. Hence, the difference between these two species is predicated on the width/expansion of the lemmas and paleas, also resulting in a visible difference of phytolith morphology at the center of lemmas and paleas where silicified epidermal long cells are most complex, but can be differentiated. Consequently, based on ΩIII phytoliths we find significant differences in the central part of lemmas and paleas between the two species (three phytolith parameters, W1-3, W2-3, and H-3 from foxtail millet, were larger than comparable ones from green foxtail). Moreover, discriminant function analysis for ΩIII-type phytoliths showed that 78.4% of 872 foxtail millet data and 76.9% of 607 green foxtail data were correctly classified. These results suggest that ΩIII phytoliths from upper lemmas and paleas are reliable tools for distinguishing these two species.

Because a phytolith is shaped almost completely according to the cell outline, three phytolith parameters, as mentioned above, reveal that the average size of undulated epidermal long cells from foxtail millet is greater (expressed as parameter W1 and W2) and the space between adjacent epidermal long cells (expressed as parameter H) is larger than those from green foxtail (Figures 4 and 5). We speculate that morphological differences in epidermal long cells between these two species is mainly due to increased grain size.

Increased in seed size is a remarkable result of selective domestication and cultivation [3], [62]. This character is close related to increased efficiency and competitiveness in germination and early growth in open, heavily disturbed soils and with deeper burial of seeds, which is expected under tillage [62], [63]. This domesticated trait alteration has already been demonstrated in many crops, including rice [4], [63], wheat, and barley [64]. Consequently, compared with green foxtail, we speculate foxtail millet has larger intercellular spaces between adjacent epidermal long cells and greater epidermal long cell size in upper lemmas and paleas mainly due to large grain size, as a result of plant domestication and cultivation.

However, little is known about the linkage between phytolith morphological differentiation and genotypes when cultivated by various traditional means. Additionally, there has some confusion in the use of this morphological indicator. About 25% data are incorrectly classified, make the identification difficult at least when phytoliths are few, and we find only one diagnostic feature to distinguish the two taxa, more local samples are needed to detect if other diagnostic features are practicable. Further research is needed on these topics.

### Clues to the place of origin of foxtail millet

To date, many genetic methods, for example, RFLPs [22], mitochondrial DNA [23], and rDNA [24], [25], reveal that foxtail millet might have multiple centers of origin. Can this hypothesis be reflected in phytolith morphology?

**Figure 9:**
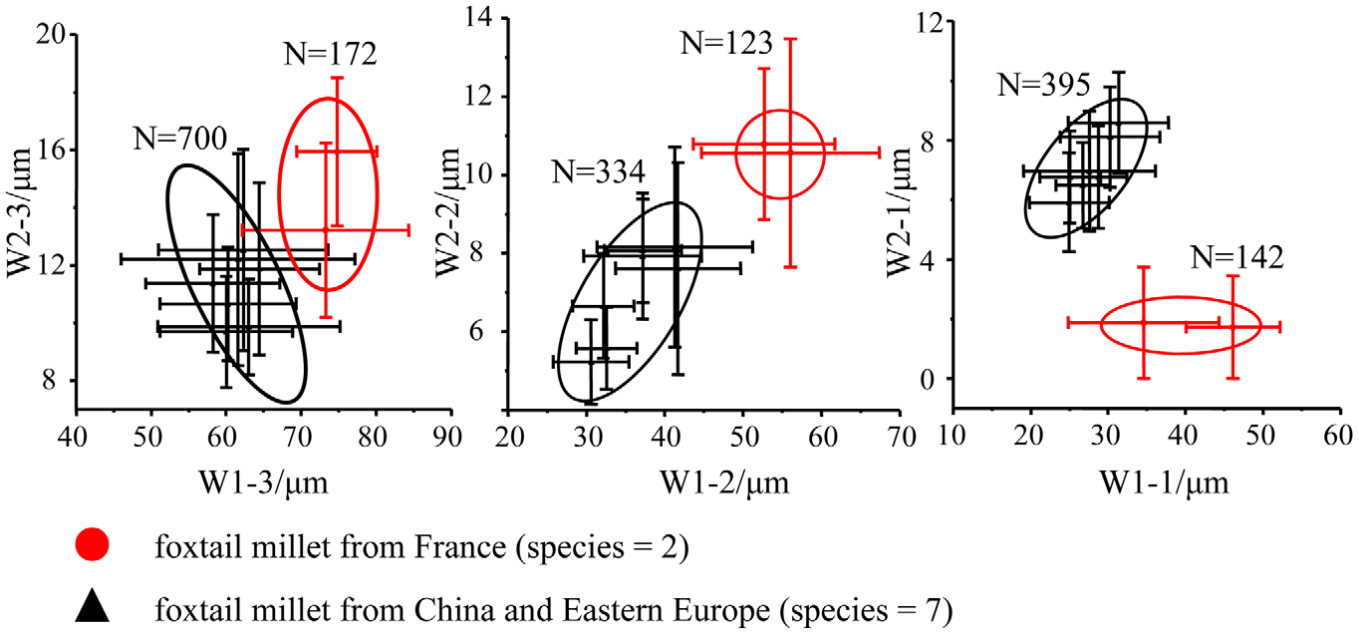
Comparison of undulated patterns of epidermal long cells for foxtail millets from China, East Europe and France. WX(X = 1, 2)-Y: W1 = width of undulated patterns of epidermal long cells; W2 = width of epidermal long cells; Y = 1, 2, 3 = ΩI, ΩII and ΩIII. N = the total number of data. Error bar = SD.

To answer this question, we dissected the spikelets of modern foxtail millets that originated in France (W28 and Z169). We found in ΩI, ΩII, and ΩIII the morphology of undulating patterns of epidermal long cells in the upper lemmas and paleas of the two samples was significantly different from millet samples that originated in China and Eastern Europe (Figure 9). These results suggest that Western European foxtail millets (W28 and Z169) have different evolutionary lineages from foxtail millets in Eastern Europe and East Asia. This primary research can also make a contribution to the key issue of millet origin. However, in this study, as samples from Western Europe are few, more foxtail millets and green foxtails from Europe and China are needed to substantiate our speculation.

Foxtail millet and green foxtail populations have rich genetic diversity and worldwide distribution [65]–[67]. The geographical diversity within both species was greater than the diversity between species (S. viridis/S. italica) [22], [68], [69]. Hence, we speculate that geographical diversity might cause the morphological difference in ΩIII-type phytolith between East Asian and Western European foxtail millets. Furthermore, foxtail millet and green foxtail are in the same family (Poaceae) and genus, and both have the same number of chromosomes (2n = 18), therefore hybridization between the two species is easier [28]. During a long period of natural evolution and domestication, continual hybridization between local foxtail millet and green foxtail may cause greater diversity of foxtail millet in different regions. Therefore, phytolith morphological difference between East Asian and Western European foxtail millet could become striking.

To eliminate the influence of values from Western European millets, we recalculated the mean values of W1, W2, and H in type ΩIII from foxtail millet and green foxtail and also performed another discriminant analysis without the foxtail millets W28 and Z169 from France. We found that the differences between the rest of the foxtail millets and green foxtails were still remarkable. For foxtail millet, W1-3, W2-3, and H-3 are about 61.3±10.9, 11.2±2.9, and 25.1±4.8 µm, respectively, and are still distinguishable from green foxtail (52.1±6.4, 8.0±1.9 , and 22.1±2.9 µm). Of the original grouped cases (foxtail millet and green foxtail), an average of 75.1% were correctly classified. This result is slightly lower than the first discriminant analysis (average = 77.7%), because we excluded the values of W1-3, W2-3, and H-3 in W28 and Z169 from France.

Although the phytolith production patterns revealed by our preliminary research give encouraging results that millet from China, Eastern Europe, and Western Europe may be different in husk phytolith morphology, more research is still needed, especially for the study of more local species from Europe. To be of practical use to investigators, further morphometric and genetic analysis of a wide variety of millet species is required. This methodology may help to understand foxtail millet domestication in future work.

### Conclusions

Foxtail millet is an important traditional crop in the Far East and other locations throughout Eurasia. Hence, to discuss its origin in early dry farming, distinguishing it from green foxtail in archaeological remains is unavoidable and vital.

In this work, new characters of phytolith identification for husks of foxtail millet and green foxtail have been developed and evaluated as a reliable way of separating the two taxa. We determined that the undulating patterns of ΩIII epidermal long cells in the upper lemmas and paleas are meaningful as criteria to distinguish foxtail millet and green foxtail. The size of single ΩIII cells from foxtail millet is larger than comparable cells from green foxtail. A discriminant function was also established: 78.4% of foxtail millet and 76.9% of green foxtail of the original grouped samples were correctly classified. The result statistically provides a robust method for distinguishing between these two taxa. Furthermore, phytolith morphology in upper lemmas and paleas from Chinese and Eastern European foxtail millets are markedly different from those of French foxtail millets, possibly suggesting that foxtail millets in Western Europe have a different evolutionary lineage from those in East Asia.

This is an attempt to distinguish between silicon structure patterns in the glumes, lemmas, and paleas occurring in foxtail millet and green foxtail. Further study is still needed, especially for morphological comparisons among local species of foxtail millets and green foxtails. Our results, if supported by additional studies of phytoliths derived from more millets and related grass species, can provide a reliable way of separating remains of foxtail millet from green foxtail based on their phytolith morphology.
